# Therapy-Induced Senescence Shapes Extracellular Matrix Niches and Fibroblast Function in Head and Neck Squamous Cell Carcinoma

**DOI:** 10.3390/cancers18071126

**Published:** 2026-03-31

**Authors:** Jetsy Montero-Vergara, Piotr W. Darski, Amy L. Harding, Keith D. Hunter

**Affiliations:** The Liverpool Head & Neck Centre, Department of Molecular and Clinical Cancer Medicine, The University of Liverpool, Liverpool L7 8XP, UK; jetsy.montero-vergara@liverpool.ac.uk (J.M.-V.); p.darski@liverpool.ac.uk (P.W.D.)

**Keywords:** cellular senescence, therapy-induced senescence, TIS, SASP, extracellular matrix, collagen architecture, cancer-associated fibroblasts, HNSCC, OSCC, oral submucous fibrosis, spatial pathology, SHG microscopy, senotherapeutics

## Abstract

Cellular senescence is a stress-induced state characterised by durable proliferative arrest and extensive transcriptional and secretory reprogramming. In cancer, senescence can suppress early tumour outgrowth, yet persistence of senescent cells and their senescence-associated secretory phenotype (SASP) may drive maladaptive inflammation, immune dysfunction, vascular perturbation and extracellular matrix (ECM) remodelling. Head and neck squamous cell carcinoma (HNSCC) provides a clinically informative context because tumours arise in injury-prone mucosa and standard therapies (radiotherapy and platinum-based chemotherapy) can induce long-lived senescent phenotypes across stromal and vascular compartments. Here, we synthesise the evidence through a signal → matrix → function framework, in which the therapy-induced SASP modules reshape collagen density, alignment, confinement and crosslinking, thereby influencing invasion, immune access, perfusion and post-treatment fibrosis. We emphasise that senescence detection in head and neck tissues is highly context-dependent and readily confounded by inflammageing, chronic mucosal injury and HPV-associated biology, necessitating a cell-type-resolved, spatially anchored, multi-axis definition that integrates growth-arrest context, nuclear/DNA damage response hallmarks and functional outputs. We highlight oral submucous fibrosis (OSF) as a matrix-primed precursor state that exemplifies convergence between chronic injury, fibrosis and senescence-adjacent programmes. Finally, we propose an integrated translational roadmap combining multiplex spatial pathology with quantitative collagen imaging to map therapy-induced senescence–ECM niches and support biomarker-guided testing of senomorphic, senolytic and matrix-normalising strategies in HNSCC.

## Simple Summary

Cellular senescence is a state where damaged cells stop dividing but stay alive and change the signals they release. In cancer, this can help in the short term by limiting early tumour growth. Over time, however, lingering senescent cells can release a mix of inflammatory and tissue-remodelling factors that may harm normal function. This is especially relevant in head and neck cancers because these tumours develop in tissues that are often injured or inflamed, and common treatments like radiotherapy and platinum chemotherapy can trigger long-lasting senescence in supportive cells such as fibroblasts. This review explains how these treatment-induced signals can reshape the tumour “scaffold” (the extracellular matrix), altering collagen structure and stiffness in ways that affect tumour spread, immune cell access, blood flow, and scarring after treatment. We also discuss oral submucous fibrosis as an example of a pre-fibrotic, high-risk environment. Finally, we outline a roadmap for using advanced tissue imaging to guide testing of new therapies that target senescent cells or normalise the tumour matrix.

## 1. Introduction

Cellular senescence is a stress-induced state characterised by durable proliferative arrest coupled to extensive transcriptional, metabolic, and secretory reprogramming [1,2]. Originally framed as a tumour-suppressive mechanism that constrains the expansion of damaged or oncogene-expressing cells, senescence is now recognised as profoundly context-dependent, with consequences spanning tissue homeostasis, ageing, fibrosis, and cancer progression [3,4,5]. Although stable arrest can restrict early neoplastic outgrowth, persistence of senescent cells and their senescence-associated secretory phenotype (SASP) can drive maladaptive inflammation, immune dysfunction, vascular perturbation and extracellular matrix (ECM) remodelling—shifting senescence from a barrier to malignancy towards a microenvironmental driver of progression [6,7]. The SASP is modular and varies with inducer, cell type and time, comprising inflammatory mediators alongside ECM-active factors such as proteases, crosslinking-associated programmes, matricellular regulators and extracellular vesicle cargo [7,8].

Senescence is also central to models of organismal ageing and fibrosis, where senescent-cell accumulation drives chronic inflammation and impaired regeneration in vivo [9,10]. In parallel, fibrotic disorders are increasingly interpreted through an “accelerated ageing” lens, in which persistent stromal activation and aberrant ECM deposition coexist with senescence-adjacent states and long-lived inflammatory signalling [11]. These converging fields highlight a key implication for head and neck oncology: senescence is not solely a cell-intrinsic fate decision, but a tissue-level programme whose consequences are shaped by stromal composition, immune surveillance and—critically—the ECM.

**Figure 1:**
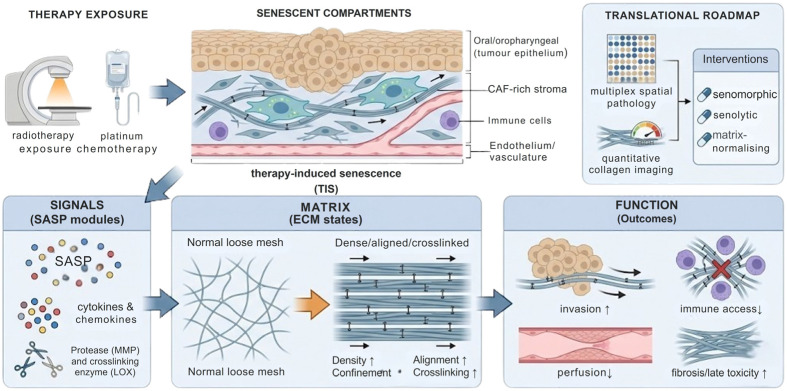
Signals to matrix to function overview of the senescence–ECM loop in head and neck cancer. Schematic summarising how radiotherapy and platinum-based chemotherapy can induce therapy-induced senescence across oral and oropharyngeal tumour epithelium and stromal compartments, including CAF-rich stroma, endothelium and vasculature, and immune cells. Senescent cells generate modular SASP outputs, including cytokines and chemokines, proteases such as MMPs, and matrix-active mediators such as SERPINE1 and LOX family crosslinking programmes, which remodel the extracellular matrix architecture from a normal loose mesh to a dense, aligned and crosslinked state with increased collagen density, confinement and stiffness. These matrix changes translate into functional consequences relevant to head and neck squamous cell carcinoma, including enhanced tumour invasion, reduced immune access, impaired perfusion, and late fibrosis and toxicity. A translational roadmap is highlighted, linking multiplex spatial pathology and quantitative collagen imaging to stratify senescence and ECM niches and to inform senomorphic, senolytic, and matrix-normalising intervention strategies.

Here, we synthesise the evidence using a signal → matrix → function framework. In this view, senescent stromal states generate SASP “modules” (signals) that reconfigure ECM composition and architecture (matrix)—including collagen density, alignment, confinement and stiffness—thereby influencing invasion, immune access, perfusion and therapy response (function) (Figure 1). The ECM governs mechanotransduction, growth factor bioavailability, and immune-cell trafficking [11,12]. Senescence and ECM remodelling are therefore reciprocally linked: ECM-derived stiffness, crosslinking and confinement can reinforce senescence persistence, while senescent stromal cells remodel the matrix through deposition, proteolysis, crosslinking-associated programmes and matricellular signalling.

Head and neck squamous cell carcinoma (HNSCC) is the seventh most common cancer globally, accounting for approximately 890,000 new cases and 450,000 deaths annually, with a five-year overall survival of approximately 50–60%; incidence is projected to surpass one million new diagnoses per year by the mid-2020s [13,14]. HNSCC, including oral squamous cell carcinoma (OSCC) and oropharyngeal squamous cell carcinoma (OPSCC), provides a clinically informative setting in which to examine senescence–ECM reciprocity because tumours arise in injury-prone mucosa exposed to chronic microtrauma, microbial challenge, mechanical stress and carcinogens, where inflammatory and wound-repair programmes are prominent. Crucially, standard-of-care radiotherapy and platinum-based chemotherapy can induce long-lived senescent phenotypes not only in tumour cells but also across stromal and vascular compartments, generating “field effects” that may persist beyond the acute injury phase [15,16]. In the head and neck, these processes matter clinically in two linked domains: oncological control and survivorship morbidity. Persistent stromal remodelling contributes to progressive fibrosis and late toxicities—including dysphagia, trismus, xerostomia and impaired upper aerodigestive function—while ECM reorganisation at tumour–stroma interfaces can shape immune access, residual disease behaviour and local recurrence risk.

The oral cavity also offers a “matrix-primed” precursor condition: oral submucous fibrosis (OSF)—in which chronic areca nut exposure drives progressive fibrosis and malignant risk [17,18]. OSF exemplifies convergence between chronic injury, fibrotic ECM remodelling, and senescence-adjacent programmes, with areca nut and smoking-associated factors inducing senescence-like states in oral fibroblasts alongside pro-fibrotic signalling such as TGF-β, consistent with a senescence–fibrosis axis in the oral mucosa [19,20,21]. Conceptually, OSF therefore serves as an instructive analogue for post-therapy fibrotic niches: both contexts feature persistent injury signalling, collagen accumulation and stiffening, and altered epithelial–stromal crosstalk that may lower the threshold for senescence persistence and reinforce matrix-skewed remodelling.

The head and neck literature provides a natural entry point for senescence-informed ECM thinking. In genetically unstable OSCC, cancer-associated fibroblast (CAF) populations have been reported to contain a higher proportion of senescent cells and to exhibit deregulated autophagy, linking stromal senescence to impaired proteostasis and altered matrix-regulatory capacity—mechanisms plausibly relevant to durable ECM remodelling and the persistence of tumour-permissive stromal niches [22]. Beyond exposure-associated senescence, therapy-induced senescence (TIS) is increasingly positioned as a clinically relevant mechanism in HNSCC: persistent TIS-associated SASP may remodel the tumour microenvironment in ways that sustain fibrosis, perturb vascular function and generate immunosuppressive or immune-excluded niches, with implications for recurrence and late toxicity [7].

Despite these advances, major evidence gaps constrain translation in HNSCC. Spatially resolved mapping of senescence markers alongside quantitative ECM architecture remains limited, leaving unresolved how senescent fibroblasts, endothelium, immune subsets and tumour cells are organised across tumour cores, invasive fronts and post-therapy margins, and how these distributions relate to collagen topology and matrix-gated immune access [23]. Although mechanobiology and fibrosis research implicates stiffness and crosslinking as drivers of senescence stabilisation, operative thresholds and causal pathways in oral fibroblasts remain poorly defined in clinically representative systems [24]. Finally, while senomorphic, senolytic and matrix-normalising strategies are increasingly plausible, biomarker-guided evaluation in HNSCC cohorts—anchored to spatially defined TIS–ECM niches—remains underdeveloped. Accordingly, this review focuses on reciprocal coupling between senescence and ECM remodelling in oral and head and neck cancers and proposes an integrated translational roadmap combining multiplex spatial pathology with quantitative collagen imaging to map TIS–ECM niches and support rational testing of senescence-modifying and matrix-directed interventions.

This review focuses on stromal fibroblast senescence and ECM remodelling in mucosal HNSCC. We do not address immunotherapy-induced senescence, non-epithelial head and neck tumours (e.g., salivary gland, thyroid), or senescence programmes confined to the malignant compartment. This is an integrative, hypothesis-building narrative review, not a systematic review. Literature was identified through structured searches of PubMed/MEDLINE and Scopus using key terms including cellular senescence, therapy-induced senescence, SASP, extracellular matrix, collagen, cancer-associated fibroblasts, HNSCC, OSCC, oral submucous fibrosis, senotherapeutics, and spatial pathology, with no language restriction. The search prioritised publications from 2010 to 2026, with inclusion of earlier seminal studies where they underpin current conceptual frameworks. Both preclinical and clinical data were included; references were selected based on mechanistic contribution, methodological quality, and relevance to the signal → matrix → function framework developed herein.

## 2. Defining Senescence in Human Head and Neck Tissues: Composite Biomarkers, Spatial Context, and Limitations

### 2.1. Context-Dependent Interpretation of Senescence Markers

A recurring challenge in translating senescence biology into pathology is that many ‘canonical’ markers are not specific to durable senescence in vivo. This problem is amplified in head and neck tissues, where high baseline epithelial turnover, periodontal inflammation, chronic micro-injury, microbial challenge, and treatment-associated DNA damage responses can all generate overlapping phenotypes. In practice, transient checkpoint arrest (e.g., stress-inducible p21), inflammatory activation (e.g., IL-6/IL-8/MMPs), and stress-associated p16 expression may occur without a stable senescent state. Consequently, single-marker inference risks conflating persistent senescence with acute injury biology or chronic inflammatory remodelling.

Consistent with current guidance, no single marker is sufficiently specific in clinical sections; senescence identification therefore requires a combinatorial strategy that integrates growth-arrest context, nuclear/chromatin or DNA damage response (DDR) persistence, and functional output, ideally anchored to lineage and spatial compartment [25]. In HNSCC, misclassification can inflate “senescence burden” in tissues dominated by inflammation, HPV-associated epithelial biology, or early post-treatment injury responses, and can distort causal claims about stromal remodelling [26].

A further, increasingly relevant limitation is that transcriptomic “senescence scores” can be sensitive to cellular composition and to overlapping stress/secretory programmes, particularly in inflamed mucosa and CAF-rich stroma [27]. This reinforces the need to treat computational scoring as supportive rather than definitive unless it is anchored to compartment-resolved biology and corroborated by orthogonal hallmarks [28].

### 2.2. A Practical Composite Framework for Tissue-Based Senescence 

**Figure 2:**
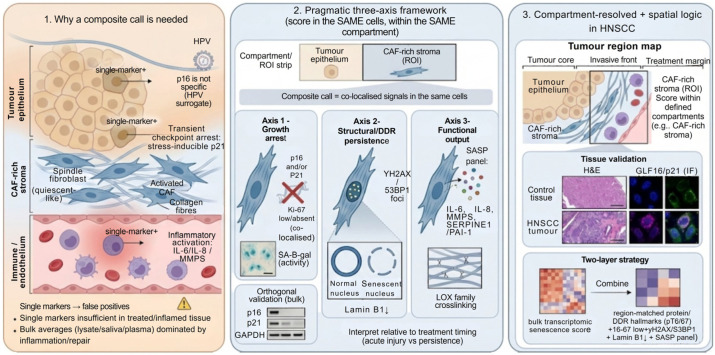
Schematic illustrating a pragmatic approach to define senescence in patient tissues. Panel 1 highlights single marker vulnerability to false positives in head and neck tissues, where p16 can reflect HPV related biology, p21 can mark transient stress, and inflammatory programmes can dominate bulk readouts. Panel 2 presents a three-axis framework comprising growth arrest, supported by concordant p16 and or p21 induction with reduced proliferation and increased SA-βgal activity. Structural and DNA-damage response support is indicated by persistent γH2AX and 53BP1 foci with Lamin B1 depletion and nuclear reorganisation interpreted relative to treatment timing, and functional output based on panel level SASP evidence including cytokines, proteases such as MMPs, and matrix-active mediators such as SERPINE1 together with LOX family-associated crosslinking programmes. Panel 3 emphasises compartment-resolved and spatial logic in HNSCC, advocating region and compartment-specific scoring across tumour core, invasive front, and treatment margins, and demonstrating tissue level validation using the GLF16 lipofuscin-associated signal with p21 in control tissue versus HNSCC. A two-layer strategy is shown in which transcriptomic senescence detection is combined with region-matched protein and DDR hallmarks to strengthen inference, particularly in treated tissues where DDR markers alone may reflect acute injury.

For reproducible tissue-based assessment—particularly in HNSCC specimens where confounding stress and repair programmes are common—a pragmatic three-axis framework can be implemented within standard pathology workflows and extended to multiplex/spatial platforms (Figure 2). The aim is to make a co-localised senescence read within a defined compartment (e.g., CAF-rich stroma), rather than scoring marker positivity across heterogeneous tissue [29]. 

A workable approach is: 

Growth arrest: at least two concordant indicators (typically p16^INK4a^ and/or p21^CIP1/WAF1^) paired with reduced proliferation (Ki-67 low/absent) in the same cells.

Structural/DDR support: nuclear or DDR hallmarks consistent with persistent damage signalling and chromatin/nuclear reorganisation (e.g., Lamin B1 depletion; persistent γH2AX/53BP1 foci), interpreted explicitly in relation to treatment timing. 

Functional output: SASP evidence based on panels rather than a single mediator, ideally including ECM-relevant outputs (e.g., MMPs, SERPINE1/PAI-1) and, where justified by context, broader matrix-remodelling/crosslinking programmes (e.g., lysyl oxidase family activity), with assignment to the correct compartment (tumour epithelium versus CAF-rich stroma versus endothelium) [1,6,8]. 

Crucially, in HNSCC this framework is most informative when applied spatially and cell-type-specifically. In head and neck tissues, bulk averages (tumour lysate, saliva, plasma) can be dominated by non-senescent inflammatory or repair states [30], masking whether putative senescence programmes are truly persistent, where they are located (tumour core vs. invasive front vs. treatment margin), and which lineage is responsible. Where spatial transcriptomics is available, composite inference can be strengthened by combining (i) a robust transcriptomic senescence detector with (ii) region-matched protein/DDR hallmarks—rather than treating either modality as sufficient alone [27]. This “two-layer” strategy is particularly useful for treated tissues, where DDR markers may reflect acute injury if not paired with persistence and module-level transcriptional evidence [31,32].

### 2.3. Growth-Arrest Markers: Necessary, but Not Sufficient

p16^INK4a^ and p21^CIP1/WAF1^ are widely used markers of growth arrest, commonly paired with low Ki-67. However, both can be induced outside stable senescence programmes; p21 can often reflect reversible stress responses and transient checkpoint enforcement [26]. Growth arrest should therefore be treated as supporting evidence rather than a stand-alone criterion, especially in tissues with active inflammation or recent cytotoxic exposure.

This caution is amplified in head and neck disease because p16 immunopositivity is biologically confounded by HPV-driven RB pathway disruption, particularly in oropharyngeal carcinoma where p16 is routinely used as a surrogate marker of HPV-associated oncogenesis rather than senescence [33]. Even outside HPV-positive tumours, p16 positivity can occur in inflamed oral mucosa and oral premalignant disorders (OPMD) that do not straightforwardly equate to durable senescence, reinforcing the need for confirmatory hallmarks [29]. P16-positive cells also accumulate in ageing and chronically stressed tissues, including sun-exposed human skin where p16^INK4a^ positivity increases with age and correlates with photoaging features, emphasising that p16 alone does not establish a stable senescence programme [34,35].

An additional tumour-specific complication in HNSCC is frequent disruption of TP53, particularly in HPV-negative disease. As p21 is classically downstream of p53, tumour-cell stress responses may decouple p53–p21 checkpoint wiring from durable arrest, and stress may instead resolve through alternative outcomes or reliance on other checkpoint mechanisms. Practically, this strengthens two points relevant to tissue interpretation: (i) p21 should not be used as a universal “senescence marker” in TP53-malignant epithelium without persistence hallmarks, and (ii) stromal scoring should be prioritised, since fibroblasts/endothelium can still enter long-lived therapy-induced senescent states that remain biologically consequential [1].

Where tissue constraints allow, inference is strengthened by including markers of cycling capacity or replication licencing (e.g., MCM/PCNA context) but the minimum principle should be co-localisation of ≥2 arrest features in the same cells, scored with pre-specified thresholds rather than bulk positivity [25,26].

### 2.4. Nuclear and DDR Hallmarks: Increasing Specificity in Clinical Sections

Lamin B1 depletion is frequently used as a marker of senescence-associated nuclear and chromatin reorganisation and is most informative when interpreted alongside growth-arrest features rather than in isolation [1,36]. DDR-associated features such as persistent γH2AX and 53BP1 foci can increase confidence but are not unique to senescence and may also reflect acute post-therapy damage. Interpretability therefore depends on treatment timing and on concordance with additional hallmarks of persistence.

Where feasible, more discriminating DDR-linked approaches include the assessment of telomere-associated DNA-damage foci, which may better reflect chronic DDR maintenance because such lesions can be difficult to repair [37,38,39]. For HNSCC cohorts, explicit documentation of the sampling interval relative to therapy is essential to separate acute injury biology from persistent senescence-associated DDR.

Additional tissue-compatible structural readouts can further support a senescence call when nuclear/DDR features are equivocal. One example is GLF16, a fluorophore-conjugated Sudan Black B analogue that labels senescence-associated lipofuscin aggregates and enables rapid detection by fluorescence microscopy and flow cytometry, including in tissue contexts [40,41]. In practice, this is particularly convenient for tissue microarrays (TMAs), where the GLF16 signal can be scored across many cores with consistent acquisition settings and combined with multiplex lineage markers. As lipofuscin can also accumulate in chronic stress states, GLF16 is most defensible as an adjunct readout, interpreted alongside lineage anchoring and the composite hallmarks above rather than as a stand-alone criterion [42].

### 2.5. Functional Readouts: SASP Modules and ECM-Active Programmes

As senescent cells remain viable and metabolically active, SASP provides a functional window into senescent state. However, SASP is modular and context-dependent—varying with inducer, tissue, cell type, and time—so interpretation is most robust when based on panels rather than single mediators [6,8,43]. For head and neck translation, SASP assessment should extend beyond inflammatory cytokines to include ECM-remodelling outputs, particularly in CAF-rich regions where senescence–matrix feedback is likely to be most consequential.

Importantly, SASP is not a discrete “senescence-only” secretome: it overlaps substantially with secretory programmes of activated fibroblasts/CAF states induced through other routes, including TGF-β-driven myofibroblastic activation and inflammatory CAF programmes. This means that cytokines/proteases alone cannot establish senescence in tissue. A defensible interpretation requires senescence context (durable arrest plus persistence hallmarks) together with module structure and spatial attribution. In practice, this reduces the risk of labelling non-senescent-activated CAF biology as “senescence” simply because IL-6, CXCL8, MMPs, or SERPINE1 are elevated in fibrotic or inflamed stroma. 

Novel and experimental readouts that can complement soluble SASP panels include profiling of extracellular vesicle-associated cargo, proteomic scoring of SASP modules, and transcriptomic signatures that assign secretory programmes to specific lineages (for example, distinguishing inflammatory outputs from ECM-skewed secretory states within CAF-rich stroma versus tumour epithelium or endothelium) [44]. These approaches are particularly valuable in HNSCC where compartment mixing is a dominant source of interpretive error. Gene-score-based or module-based senescence annotation at single-cell resolution is increasingly used to support lineage attribution but still requires spatial anchoring in solid tissues where senescent cells are not uniformly distributed [27].

### 2.6. Spatial Attribution: Why Localisation Matters in HNSCC and OSF

Senescence in vivo is rarely uniform. In solid tumours and fibrotic oral disease, senescent-like states may cluster within stromal niches, treatment-altered margins, perivascular regions or fibrotic compartments. Bulk transcriptomic or proteomic readouts (tumour lysate, plasma, saliva) are therefore intrinsically limited for assigning which cells are senescent and where they sit relative to ECM architecture. This limitation is especially acute for the central thesis of this review: collagen topology and matrix “gating” of immune access are inherently spatial phenomena, so senescence and SASP signals must be interpreted within the same tissue architecture, using compartment-resolved and spatially anchored readouts. Multiplex and spatial approaches increasingly allow co-mapping of: (i) senescence markers, (ii) lineage markers (tumour, CAF-rich stroma, endothelium, immune), and (iii) ECM features (collagen topology, matricellular deposition) within the same regions of interest [29]. This is also directly relevant to OSF as a matrix-primed, fibrosis-rich precursor state, where chronic injury and fibrotic remodelling can generate senescence-adjacent patterns that are difficult to separate without cell-type and spatial anchoring.

Additional OSF-focused evidence strengthens this point. Direct support for senescence-like programmes in OSF fibroblasts comes from areca nut alkaloid exposure studies in oral fibroblasts, in which senescence was assessed using senescence-associated beta-galactosidase activity, persistent 53BP1 DNA-damage foci, increased p16INK4a, and reduced Ki67, together with secretory readouts showing increased TGF-β and a smaller increase in MMP-2 [45]. These features are consistent with a durable senescence-like fibroblast state rather than transient activation alone. Complementing this, human OSMF tissue studies have identified p16INK4a-positive fibroblastic cells together with elevated IL-6, VEGF, MMP-9, and LOX, linking fibroblast senescence-associated secretory and matrix-remodelling programmes to a fibrosis-rich clinical lesion [46]. Earlier fibroblast work showing increased TIMP-1 and TIMP-2 secretion in OSF further supports the concept that an altered fibroblast state and impaired matrix turnover are integral to disease biology [47].

These observations help distinguish, but also connect, senescence-like fibroblast states and classical myofibroblast activation in OSF. Myofibroblastic transition in buccal mucosal fibroblasts has been demonstrated using alpha-SMA expression, stress fibre formation, collagen-gel contraction, migration and wound-healing assays, and ZEB1-dependent transcriptional activation after arecoline exposure [48]. In contrast, the senescence-oriented studies emphasise growth arrest, DNA-damage persistence, and SASP-like secretory output, rather than contractility per se [45,46]. The overlap is nevertheless biologically important: both programmes converge on TGF-beta, LOX, MMPs, and matrix remodelling, making OSF a useful oral model of a mixed fibrotic–senescent stromal niche rather than a purely myofibroblastic lesion [45,46,47,48].

Clinical evidence also supports the relevance of OSF as a fibrosis-associated precursor state with malignant potential, although direct senescence-to-outcome studies remain limited. Systematic reviews and meta-analyses estimate malignant transformation rates in the range of approximately 4% to 6%, with epithelial dysplasia conferring higher risk [49,50]. Histomorphometric and polarisation studies further suggest that collagen organisation changes as disease advances: collagen fibres become thicker, more densely packed, and increasingly reorganised, and birefringence patterns have been linked to histopathological stage and degrees of epithelial dysplasia, supporting the idea that matrix architecture evolves alongside neoplastic risk [51,52]. At present, however, these data do not establish a validated senescence-specific or collagen-architecture biomarker for malignant transformation, and that limitation should be stated explicitly.

Where OSF progresses to OSCC, retrospective clinical series suggest that the resulting tumours may show distinct clinicopathological behaviour, including younger age at presentation, better differentiation, lower nodal burden, and in several cohorts better disease-free or oncologic outcomes than OSCC arising without OSF [53,54]. These studies are informative because they indicate that the pre-existing fibrotic stromal context of OSF is biologically consequential rather than incidental. At the same time, direct clinical studies linking senescent fibroblast burden in OSF to treatment response in OSCC patients remain lacking. Accordingly, the strongest interpretation at present is that OSF provides a clinically relevant, spatially anchored human model in which chronic injury, fibroblast-state change, collagen remodelling, and malignant potential can be examined together in situ, while direct outcome correlations for senescent stromal subsets remain an important evidence gap [45,46,49,50,51,52,53,54].

### 2.7. Technical Considerations for Clinical Material

Even appropriate marker selection can fail if pre-analytical variables are not controlled. Immunohistochemistry is sensitive to cold ischaemia time, fixation type and duration, antigen retrieval conditions, antibody selection, and storage; each of which can materially affect staining intensity and comparability across cohorts [55]. For studies comparing pre- and post-therapy specimens or multi-centre collections, systematic documentation of these variables is essential for interpretability and reproducibility.

Where possible, analytical robustness is strengthened by: (i) using matched controls within the same batch, (ii) quantifying signal with pre-defined thresholds and consistent acquisition settings, (iii) scoring co-localisation explicitly (rather than separately scoring markers in different compartments), and (iv) reporting therapy timing (weeks/months post-RT/chemo) alongside staining results. These steps are particularly important for DDR-linked markers, which are highly sensitive to acute treatment effects.

### 2.8. Limitations and Evidence Gaps

Clinically actionable senescence detection in head and neck disease remains constrained by limited validation of multi-marker panels optimised for oral tissues and by frequent reliance on single surrogate readouts vulnerable to confounding by inflammation, HPV-associated p16 biology, altered tumour-cell checkpoint wiring, and acute treatment effects. Spatial mapping of senescent subsets remains sparse, and senescence burden is only rarely linked quantitatively to ECM features such as collagen alignment, fibre density, or desmoplastic architecture—properties likely to matter most for invasion and immune access [56]. Finally, therapy-induced signatures (TIS) have not been systematically benchmarked in longitudinal, well-timed HNSCC cohorts, limiting biomarker-driven stratification for senescence-modifying and matrix-normalising interventions [57,58,59].

Methodologically, two gaps stand out: (i) the field lacks widely adopted “gold-standard” negative controls for senescence in complex human tissues, and (ii) many scoring approaches remain sensitive to heterogeneity across senescence inducers, tissues, and platforms—driving interest in universal scoring and deep-learning approaches that generalise across contexts [28]. Tools trained to detect senescent cells in both single-cell and spatial transcriptomics can accelerate hypothesis generation, but they still require orthogonal validation in the specific tissue and treatment setting under study [27].

Taken together, these constraints support a central conclusion: in HNSCC, senescence is best interpreted as a tissue-organised programme, evaluated through compartment-anchored composite definitions and their spatial relationship to ECM organisation and matrix-active outputs.

## 3. The Senescence-Associated Secretory Phenotype: A Clinically Ambiguous, Matrix-Modulating Programme

### 3.1. Composition, Regulation, and Functional Polarity

The defining functional feature of senescence beyond growth arrest is the SASP: a heterogeneous output of cytokines, chemokines, growth factors, proteases, extracellular vesicles, lipids, and matrix-modifying enzymes [6,60] (Table 1). Conceptually, senescence comprises both an output layer, secretory programmes that act on neighbouring cells and matrix, and a persistence layer, sustained by maintenance and survival programmes. SASP transcriptional control is regulated by convergent stress and inflammatory pathways, including p38–NF-κB and C/EBPβ, mTOR-linked inflammatory reinforcement, and innate immune sensing circuits such as cGAS–STING, which couple persistent damage signalling to inflammatory transcriptional programmes [61,62]. Senescent cells frequently display apoptosis resistance and survival dependencies, including reliance on BCL-2 family anti-apoptotic proteins, which underpins the rationale for selective senolytic strategies in some settings [63]. 

In acute settings, SASP can contribute to tumour suppression by reinforcing paracrine arrest and promoting immune recruitment and clearance of damaged cells [74]. In chronic settings—ageing, fibrosis, and therapy-altered tissues—persistent SASP becomes maladaptive: it sustains inflammatory tone, remodels local immune composition and function, promotes angiogenesis and epithelial plasticity, and re-engineers the extracellular niche in ways that can facilitate tumour progression and fibrosis-associated morbidity [6,7]. In head and neck disease, this “time-dependent polarity” is particularly important because standard therapies can create durable stromal injury fields in which secretory programmes persist beyond tumouricidal intent.

### 3.2. Interpreting SASP in HNSCC: Intrinsic Ambiguity

For HNSCC, the key translational problem is that SASP is not senescence-exclusive. Many SASP components overlap substantially with secretomes of activated fibroblasts and CAF states induced by non-senescent routes, including TGF-β-driven myofibroblastic activation and inflammatory CAF programmes [75]. Second, SASP is not static: composition and intensity vary with inducer (replicative, oncogene-induced, therapy-induced), cell type, and time after induction, complicating inference from individual mediators and challenging reductionist therapeutic targeting.

A second ambiguity is cell state mixing over time. Following therapy or chronic injury, tissues can contain a mosaic of transiently stressed cells, activated CAFs, and genuinely senescent populations, each contributing overlapping secreted factors. This mosaic is expected to vary across tumour core, invasive front, and post-treatment margins, and is further shaped by hypoxia, acidosis, and local matrix mechanics. These features collectively mean that SASP should be treated as a context-dependent, matrix-modulating programme rather than a discrete biomarker class.

### 3.3. CAF-Derived SASP as a Matrix-Remodelling Driver in Oral Disease and HNSCC

Fibroblasts—particularly CAFs—are prominent sources of SASP-like signalling and frequently exhibit ECM-oriented secretory outputs, including proteases and matrix-regulatory programmes that can reshape collagen organisation and tissue mechanics [7,11]. This is especially relevant in OSF and OSCC/HNSCC, where fibrosis and ECM remodelling are central biological features. Therapy-induced senescence can accumulate in stromal compartments after radiotherapy and chemotherapy, plausibly extending matrix-active SASP programmes into durable post-treatment niches [15,16].

Single-cell and spatial studies in OSCC/HNSCC resolve CAF heterogeneity into states with distinct secretory and mechanical behaviours. ECM-remodelling CAF programmes enriched for COL1A1/COL1A2, FN1, and matricellular genes such as POSTN (often described as eCAFs or ECM-CAFs) align with matrix-depositing compartments, while myofibroblastic CAF states (myCAFs) marked by ACTA2, TAGLN and MYH11 align with high-tension stroma capable of promoting collagen alignment and stiffness [76,77,78,79,80,81]. Many datasets also identify an “activated” myofibroblast programme enriched for markers such as LRRC15 and/or COL11A1, repeatedly associated with invasive ECM remodelling and immunosuppressive niches across epithelial cancers [76,79,82,83,84,85]. Inflammatory/chemokine CAF programmes (iCAFs) expressing IL6, CXCL8 with broader chemokine networks can amplify remodelling indirectly via protease induction and immune reprogramming [78,79,86,87]. Antigen-presenting CAF states (apCAFs; CD74/MHC class II expression) have been reported across tumour types and may contribute to immune modulation through non-canonical pathways [56,80,88].

Additional fibroblast states, including interferon-responsive modules, cycling fibroblasts (often transient), and perivascular/pericyte-like stromal populations, may complicate interpretation if vascular-associated stroma is not clearly separated from matrix-remodelling fibroblasts [81,89]. In HNSCC, spatial and single-cell studies have also linked specific CAF states to reduced CD8 T-cell infiltration and impaired anti-tumour activity, reinforcing the concept that CAF state and location jointly determine functional impact on tumour behaviour and treatment response [90,91,92,93,94]. Importantly, the boundaries between these CAF programmes are not rigid: plasticity between eCAF, myCAF, and iCAF states has been reported in response to changing microenvironmental cues. Senescence may represent an additional axis of CAF reprogramming in which prolonged genotoxic or inflammatory stress locks fibroblasts into durable secretory states that overlap with—but are not identical to—canonical CAF activation programmes [75,90,95]. Whether therapy-induced senescence preferentially arises within specific CAF subsets, or whether it represents a convergent endpoint accessible from multiple starting states, remains an open question with direct implications for stromal targeting strategies in HNSCC.

## 4. ECM Mechanics and Architecture as Upstream Regulators of Senescence 

### 4.1. Bidirectional Coupling Between Senescence and ECM Remodelling

While preceding sections addressed SASP as output, the ECM also functions upstream as a regulator of senescence induction and persistence. Matrix biochemical composition and biomechanics shape cell fate through pathways converging on cytoskeletal tension, nuclear deformation, DNA damage signalling, chromatin organisation, and mechanosensitive transcriptional programmes [70,96]. This supports a bidirectional loop: senescent stromal cells secrete ECM-active SASP modules that alter collagen deposition, organisation, and crosslinking, while the remodelled matrix reinforces stress signalling and stabilises senescence programmes in neighbouring stromal and epithelial compartments [24,97,98].

In head and neck tissues, this reciprocity is especially relevant after cytotoxic exposure, where the mechanical consolidation of a treated field (stiffness, alignment and confinement) can outlast acute injury and contribute to long-lived stromal phenotypes with consequences for fibrosis, immune access and residual disease ecology [99,100].

### 4.2. Matrix Stiffness and Mechanotransduction (Crosslinking, LOX/LOXL, FAK–RhoA/ROCK)

Progressive collagen deposition and enzymatic crosslinking increase tissue stiffness and elevate mechanical loads experienced by resident cells [101]. Lysyl oxidase family members (LOX/LOXL) crosslinking contributes to fibril stabilisation and stiffening, promoting sustained force transmission through integrins and focal adhesions [70,71]. In stiff or highly crosslinked matrices, cells exhibit increased actomyosin tension and enhanced mechanotransduction via integrin–FAK signalling and downstream RhoA–ROCK pathways, with consequent changes in nuclear shape and nuclear-envelope strain that can engage DNA damage responses and growth-arrest programmes [70,72,96].

Across fibrotic disease and solid tumour models, persistent stiffness is associated with reinforcement of senescence-associated phenotypes, particularly in fibroblasts, whereas reduced crosslinking or disruption of mechanotransduction attenuates stiffness-dependent reprogramming [70,72,102]. In HNSCC, particularly OSCC, dense, remodelled collagen is frequently observed at tumour–stroma interfaces and within treatment-altered tissues, making stiffness-driven signalling a plausible contributor to senescence persistence, even though direct causal evidence in patient material remains limited [59,103].

### 4.3. Confinement and Collagen Topology: Senescence Reinforcing Senescent Niches Versus Invasion Tracks

Bulk stiffness alone is an incomplete descriptor of ECM ‘load’. Collagen architecture determines effective inter-fibrillar spacing, pore size, and the degree of physical confinement experienced by cells narrowing the microscopic gaps required for cell spreading, protrusion dynamics, and migration. As collagen accumulates, aligns, and becomes crosslinked, confinement increases, elevating local solid stress and restricting cytoskeletal adaptation [104,105]. Confinement-driven nuclear strain can trigger mechanosensitive stress programmes and DNA damage signalling that favour stable arrest, providing a route for architecture to stabilise senescence in stromal compartments [96,106,107,108].

**Figure 3:**
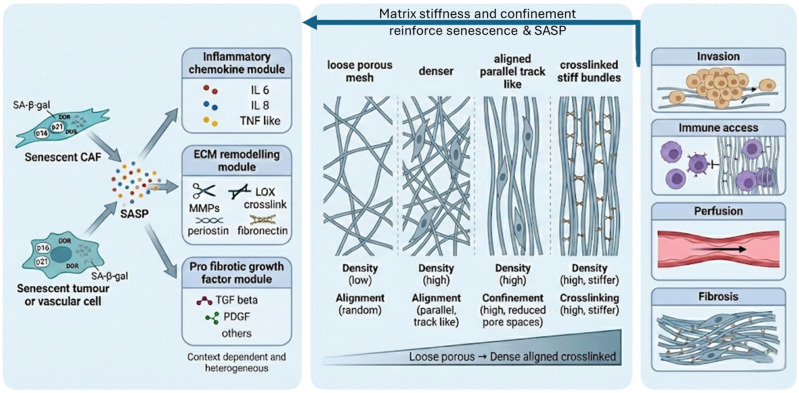
ECM mechanics and architecture as upstream regulators of senescence in HNSCC. Senescent compartments, including cancer-associated fibroblasts and senescent tumour or vascular cells, produce modular SASP programmes that include inflammatory mediators, matrix remodelling proteases, and crosslinking and growth factor signals. These outputs shift extracellular matrix architecture from a loose porous mesh toward denser, aligned, and crosslinked collagen bundles that increase tissue stiffness and confinement. In turn, stiff and confined matrices act as upstream regulators by reinforcing persistent senescence and matrix-active SASP programmes, creating a self-sustaining loop. The resulting ECM states influence tissue level consequences relevant to head and neck cancer, including enhanced invasion along aligned fibres, reduced immune access, impaired perfusion, and fibrosis with late treatment toxicity.

This produces a duality with clear translational relevance. Collagen-dense, highly confining regions may preferentially sustain persistent senescence in stromal compartments and sustain ECM-active outputs, maintaining a self-reinforcing cycle between matrix density, confinement, and senescent niche maintenance [24,102]. The same remodelling processes can generate aligned fibres that function as contact-guidance tracks exploited by tumour cells for invasion [109,110]. In HNSCC, where collagen and ECM organisation varies spatially across several niche identities, i.e., tumour cores, invasive fronts, and therapy-altered stroma, a single ECM state can therefore be simultaneously senescence-stabilising (for stroma) and invasion-permissive (for tumour cells), strengthening the need to quantify architecture rather than infer it from composition alone [111] (Figure 3).

### 4.4. Oral and Head and Neck Contexts: OSF as a Fibrotic–Senescent Precursor Niche

The oral cavity and oropharynx are exposed to repeated microtrauma, microbial challenge, and carcinogenic insults, creating tissue conditions in which wound-repair, inflammatory signalling, and fibrosis can become chronically engaged. OSF is particularly informative in this regard: chronic exposure to areca nut-associated factors promotes fibroblast activation, sustained TGF-β signalling, and excessive ECM deposition and crosslinking, producing progressive stiffening and reduced tissue compliance [46,67,112]. Senescence-associated features have been reported within this fibrotic stroma, alongside ECM alterations that disrupt epithelial–stromal crosstalk and may lower the threshold for malignant transformation [46,47].

In parallel, OSCC models support the view that tumour–stroma interactions can promote fibroblast dysfunction and senescence-like phenotypes, including proteostasis and autophagy-associated alterations reported in genetically unstable OSCC-derived CAFs [22,90]. Although mechanistic links between autophagy disruption, senescence stability, and ECM accumulation remain incompletely resolved, these observations are consistent with a broader model in which impaired matrix turnover and persistent stromal stress responses reinforce one another, sustaining a long-lived, fibrosis-prone microenvironment.

## 5. Tissue Context, Ageing, and Therapy-Induced Senescence in Head and Neck Cancer

### 5.1. Pre-Existing Tissue Context: Ageing, Inflammageing, and Mechanical Drift

TIS develops within a tissue landscape that is seldom “baseline normal” in head and neck cancer. Oral and oropharyngeal mucosa are shaped by lifelong exposure to micro-injury, microbial challenge, and chronic inflammation, and a subset of patients have pre-existing fibrosis [113,114]. Age-related changes in immune competence and ECM homeostasis shift the set-points governing whether senescence is induced, how long it persists, and how SASP signals are interpreted. This context is essential when distinguishing stable senescence from transient repair biology and when explaining why the same therapies produce divergent long-term stromal outcomes across patients [1,59,115].

Ageing is not a neutral background variable in head and neck cancer. Even before malignancy or therapy, ageing mucosa and stroma may adopt a low-grade inflammatory tone (“inflammageing”), altered matrix turnover, and changes in immune surveillance that bias tissues towards chronic remodelling rather than complete resolution after injury [113,116,117]. Practically, the challenge in head and neck specimens is therefore not whether senescence-associated markers can be detected, but whether they co-localise within the correct lineage compartment and persist in patterns consistent with durable senescence rather than transient checkpoint activation or repair responses.

With age, fibroblasts show reduced regenerative capacity and altered stress responses, while ECM homeostasis shifts towards slower turnover and increased propensity for persistent fibrosis after injury [24,118]. Across tissues, ageing is associated with changes in collagen organisation and crosslinking, altered expression of matricellular proteins, and mechanical drift towards stiffer, less permissive matrices [119]. These ECM set-points are not passive: stiffness and architecture can modulate DNA damage signalling and stress pathways, influence senescence induction and maintenance, and shape the diffusion and localisation of SASP mediators within tissue niches. In HNSCC, where tumour growth and invasion often occur in chronically inflamed or damaged mucosa, an age-shifted baseline may predispose the tumour bed to persistent, matrix-dominant remodelling after therapy [120].

The oral cavity is uniquely exposed to repeated micro-injury, periodontal disease, microbial challenge, dental procedures, and wound-repair programmes. Ageing can intensify these baseline inputs, increasing inflammatory signalling and reactive stromal activation that overlaps with elements of senescence biology [121]. This is a key reason single readouts are unreliable in head and neck tissue: IL-6/IL-8, SERPINE1/PAI-1, MMPs, or isolated p16 positivity may all increase in non-senescent injury states. Accordingly, the most defensible approach in oral tissues is compartment-anchored, multi-axis interpretation that integrates growth-arrest context, structural/DDR support, and SASP/module alignment, with explicit reference to therapy timing where relevant.

### 5.2. Immunosenescence, Vascular Dysfunction, and Matrix-Gated Immune Access

Ageing reshapes the immune landscape of the tumour microenvironment. Immunosenescence involves altered immune composition and function, including changes in T-cell competence and myeloid behaviour that modify immune surveillance and clearance capacity [122]. For senescence biology, this has two direct implications. First, impaired immune-mediated clearance can permit senescent stromal cells to persist, extending SASP exposure and ECM remodelling [123]. Second, an aged immune context may amplify maladaptive feedback: SASP signals that recruit immune cells acutely may, when sustained, preferentially support suppressive or dysfunctional populations, reinforcing chronic inflammation alongside immune suppression [124]. In head and neck tissues, where baseline inflammatory and microbial inputs are common, these effects may increase both the persistence of senescent niches and the difficulty of distinguishing them from long-standing repair programmes.

From an immune perspective, SASP outputs can be considered as modules with distinct consequences: mediators that recruit and shape myeloid populations, and mediators that govern CD8 T-cell access and function. A recurrent myeloid-oriented profile includes inflammatory cytokines, colony-stimulating factors, and chemokines that recruit monocytes and granulocytes [64]. In chronic contexts, these programmes are often linked to macrophage polarisation and recruitment of suppressive myeloid populations that constrain effective anti-tumour immunity. A complementary axis includes T-cell-recruiting chemokines and positioning cues, counterbalanced by suppressive mediators such as TGF-β [68].

Importantly, immune access is also physically gated by the matrix. ECM-active components of SASP—including MMP activity, SERPINE1/PAI-1, and crosslinking-associated programmes such as LOX/LOXL—reshape collagen organisation, stiffness, and permeability, thereby influencing whether CD8 T cells can enter and persist at tumour–stroma interfaces [125,126]. Framing SASP in this way separates immune composition from the physical permissiveness of the microenvironment—an especially important distinction in HNSCC, where stromal organisation can be a dominant determinant of immune exclusion [117].

Senescence is not restricted to fibroblasts; vascular compartments may act as both targets and amplifiers of chronic remodelling. Endothelial dysfunction and senescence-like programmes have been linked to impaired perfusion, barrier alterations, and pro-fibrotic signalling in multiple injury contexts [127]. In chronically fibrotic oral disease, vascular alterations have been described alongside stromal fibrosis, providing a plausible route to tissue hypoxia and aberrant angiogenesis that can further reinforce pro-fibrotic and immune-modulating cues [128]. Hypoxia can promote matrix remodelling programmes and reshape immune behaviour, tightening the link between stromal senescence, ECM architecture, and immune escape [129].

### 5.3. Induction and Persistence of Therapy-Induced Senescence

**Figure 4:**
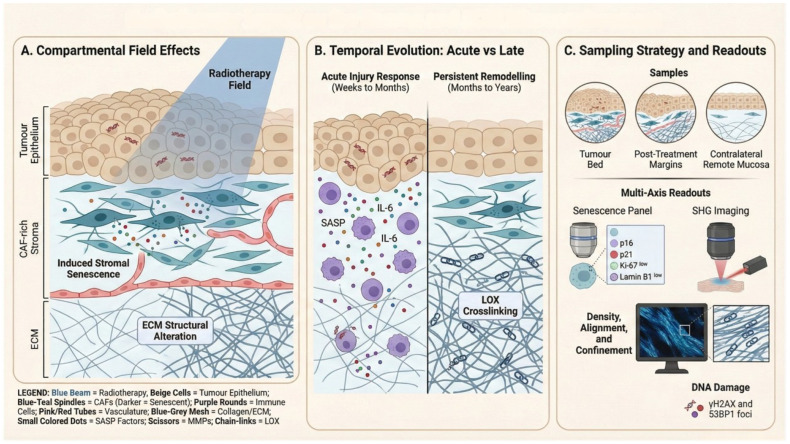
Radiotherapy field effects, temporal evolution, and sampling design for therapy-induced stromal and matrix remodelling in HNSCC. A radiotherapy field can span oral or oropharyngeal epithelium, vasculature, immune cells, and CAF-rich stroma, creating field effects across compartments rather than a single lesion-restricted response (**A**). The response evolves over time from an early, injury-associated phase dominated by cytokine-rich SASP and DNA damage signalling, to a later, persistent remodelling phase enriched for matrix-active programmes including MMP activity and LOX-mediated crosslinking, accompanied by consolidation of collagen density, alignment, stiffness, and confinement (**B**). A compartment and time aware sampling strategy compares tumour bed, post treatment margins, and contralateral or remote mucosa, integrating a multi axis senescence panel with quantitative ECM architecture readouts such as SHG collagen imaging to enable spatial co localisation and inference of clinically relevant tissue states (**C**).

Radiotherapy and platinum-based chemotherapy remain central to the management of HNSCC. Although intended to eradicate malignant cells, these modalities impose substantial genotoxic and oxidative stress across the wider treatment field, affecting tumour epithelium as well as fibroblast-rich stroma, vasculature and infiltrating immune populations [130,131]. In surviving cells, unresolved DNA damage signalling and stress pathway activation can convert an acute damage response into therapy-induced senescence (TIS): durable growth arrest coupled to sustained secretory activity that can persist beyond the window of overt inflammation and contribute to long-lived stromal reprogramming [15,16,132]. This persistence framework is clinically relevant in head and neck survivorship because late effects, including progressive fibrosis and vascular dysfunction, evolve over months to years and overlap mechanistically with chronic wound-repair programmes and senescence-linked secretory outputs [59,132] (Figure 4).

For radiotherapy, the translational issue is less beam physics than compartmental exposure and durability: conformal techniques still deliver meaningful dose to non-malignant stroma and vascular structures within the treated volume, creating conditions in which fibroblasts and endothelial cells can survive in a persistently altered state and maintain SASP signalling that is plausibly enriched for matrix-remodelling and pro-fibrotic modules [59,133,134]. Differences between photon-based approaches (e.g., IMRT/VMAT) and proton therapy may modify the spatial extent of normal-tissue exposure through reduced exit dose, but direct evidence that modality selection differentially imprints long-term senescence burden and SASP composition in HNSCC patient tissues remains limited [135,136].

Chemotherapy warrants explicit consideration as an independent driver of senescence biology. Platinum agents introduce DNA adducts and crosslinks that activate DDR networks and can promote stable arrest phenotypes in both malignant and non-malignant compartments, while also selecting for stress-adapted survivors with altered secretory and survival programmes [16,130]. In combined-modality regimens, concurrent cisplatin and radiotherapy may therefore provide layered pressures that increase the probability of persistent senescence-like states in stromal and vascular lineages, even when tumour control is achieved. Senescent-cell persistence is further supported by apoptosis resistance and context-specific pro-survival dependencies that have motivated senolytic strategies targeting BCL-2 family wiring, with several models implicating BCL-xL dependence over BCL-2 [137,138]. Preclinical studies combining DNA damage-inducing therapy with senolytic or DDR-targeting agents (e.g., navitoclax; PARP inhibition) support the feasibility of this approach, although robust validation in HNSCC cohorts and treated-field tissues is still needed [139,140].

Mechanistically, DDR signalling (ATM/ATR–CHK) and inflammatory stress programmes (including p38 MAPK–NF-κB) provide convergent routes to enforce durable arrest and sustain SASP output following genotoxic therapy [141,142]. The biological cost of persistence is increasingly viewed as microenvironmental: sustained SASP can maintain inflammatory tone, disrupt vascular function, and drive ECM remodelling that consolidates fibrosis and may generate adaptive niches for residual disease [59,143]. Evidence for prolonged senescence-associated marker expression in irradiated mucosa and tumour-adjacent tissues months to years after treatment is consistent with this model, yet most studies employ limited marker panels and provide insufficient spatial assignment to specific stromal lineages [132]. As a result, the relative contributions of tumour-cell versus stromal/vascular TIS to long-term ECM remodelling, immune access and clinical late effects remain incompletely defined in HNSCC.

### 5.4. Stress-Associated Senescence as a Continuum with TIS: OSF as an Analogue Niche

Alongside therapy-induced states, senescence-like programmes may arise in untreated mucosa and in pre-malignant disease through chronic injury, inflammation, and oxidative stress. OSF provides a clinically relevant example in which persistent irritant exposure drives sustained fibroblast stress responses that intersect with senescence-associated biology and fibrotic ECM remodelling [17,20,45,46]. Pro-fibrotic and inflammatory mediators—including sustained TGF-β pathway activity and protease-linked remodelling—support a model in which senescence-adjacent stromal states can co-exist with fibroblast activation, contributing to progressive collagen accumulation, reduced compliance, and a microenvironment permissive for epithelial dysplasia and malignant evolution [144].

From this perspective, TIS and stress-associated senescence are usefully viewed as points along a broader spectrum of stromal ageing in oral and head and neck tissues rather than as discrete entities. This framing links therapy-associated fibrosis and survivorship morbidity with pre-malignant fibrotic disease through shared pathways of persistent stromal reprogramming and ECM consolidation.

### 5.5. Compartment Sensitivity and How Senescence Maps to ECM Behaviour

A compartment-resolved view of therapy-induced senescence (TIS) is particularly informative in HNSCC because senescence in different lineages produces distinct microenvironmental consequences. In CAF-rich stroma, fibroblasts are highly stress-responsive and can adopt durable senescent phenotypes following irradiation and/or chemotherapy, with sustained secretory outputs that are disproportionately relevant to ECM remodelling. Depending on baseline fibroblast state and microenvironmental cues, stromal TIS may express programmes that appear cytokine-dominant (inflammatory signalling) and/or matrix-dominant (protease activity, matricellular signalling, altered deposition and organisation), but both routes can converge on collagen remodelling, fibre reorganisation and changes in tissue mechanics that condition invasion routes and local cell behaviour [145,146].

Endothelial and perivascular compartments represent a second axis of sensitivity. Therapy-associated senescence-like endothelial states have been linked to impaired barrier function, altered perfusion, inflammatory activation and pro-fibrotic signalling in injury contexts, offering a plausible mechanism by which vascular dysfunction can amplify fibroblast activation and collagen deposition within the treated field [127]. These vascular changes can also reshape immune trafficking by modifying adhesion, permeability and oxygenation, which in turn influences stromal remodelling loops.

Immune compartments influence TIS indirectly through surveillance and clearance capacity. Senescent-cell persistence is shaped by NK- and CD8-mediated recognition of stress ligands and by the balance of pro-clearance versus suppressive immune states [147,148]. In HNSCC, terminally differentiated/senescence-adjacent T-cell phenotypes (e.g., CD28^−^CD57^+^ populations) are detectable in blood and tumour material and may modulate inflammatory tone even when distinctions from exhaustion remain context-dependent [149,150]. Taken together, the compartmental model motivates a practical translational inference: durable, matrix-relevant consequences are most likely when fibroblast and vascular TIS persist in proximity and are not effectively cleared, rather than when senescence-like markers are detected transiently in isolation.

### 5.6. SASP Heterogeneity and Temporal Evolution After Therapy

A defining feature of TIS is that SASP output is not uniform: it varies with inducer (radiation, platinum, combined modality), lineage (fibroblast, endothelium, tumour), and time after induction [6,8]. This heterogeneity undermines single-mediator interpretation and supports a module-based framing, in which secretory programmes are read as structured patterns rather than discrete factors. Temporal programming is also central: early senescence states can be shaped by Notch-associated and TGF-β-skewed secretory features, with later transitions towards broader inflammatory and tissue-remodelling outputs as damage signalling persists [1,65,151,152]. Proteomic atlases reinforce this point by showing both recurrent “core” SASP elements and strong context-specific divergence across cell types and inducers [8].

For head and neck translation, the key implication is methodological rather than speculative: post-therapy studies require time-stamping and spatial attribution. Signals measured in plasma, saliva, lysates or bulk transcriptomes can reflect acute injury, chronic remodelling, or mixed compartments, and are often insufficient to identify whether a persistent senescent stromal niche is present. The most interpretable studies will therefore pair (i) lineage-anchored composite senescence definitions in tissue with (ii) regionally defined sampling (e.g., tumour margin, normal-adjacent mucosa, fibrotic stroma) and (iii) time-locked collection relative to therapy [8,132].

### 5.7. Mechanical Dynamics and Stromal Reprogramming After Therapy

TIS unfolds within a mechanically evolving tissue landscape. Therapy initiates acute injury and repair responses that can later consolidate into remodelling characterised by increased collagen deposition, altered fibre organisation and enzymatic crosslinking—changes that increase stiffness, reduce effective inter-fibrillar spacing and elevate solid stress. In turn, mechanical consolidation can reinforce stromal stress signalling and stabilise long-lived phenotypes, consistent with a model in which the matrix is not only a downstream target of TIS but a factor that helps sustain it [24,59].

Ionising radiation can promote senescence through direct DNA damage and sustained oxidative stress, engaging DDR pathways and stress/inflammatory signalling that maintain arrest and secretory outputs in surviving stromal and vascular cells [141,142]. In head and neck regimens that expose broad anatomical fields, this creates a plausible route to persistent “treated-field” niches in which fibroblast and endothelial compartments remain chronically reprogrammed even when tumour control is achieved [59,115]. Sampling timing is therefore decisive: early windows are dominated by acute DDR and cytokine surges that are not senescence-specific, whereas later windows are more likely to capture persistent niche biology.

Finally, several signalling nodes implicated in both therapy response and stromal remodelling—such as p38 MAPK–NF-κB—provide convergent routes to sustained inflammatory transcription and ECM-active secretory output, including in contexts where classical checkpoint wiring may differ by tumour subtype [153]. These considerations reinforce a unifying translational premise for HNSCC: mechanically consolidated post-therapy stroma is a candidate “persistence habitat” for senescent stromal states, and integrated measurement of senescence markers alongside quantitative collagen architecture is required to test whether this habitat predicts long-term fibrosis, immune ecology and recurrence risk.

### 5.8. Persistence, Bystander Spread, and Extracellular Vesicle-Associated SASP

TIS is relevant to translation not only because it may persist, but also because it can propagate. Senescent cells can transmit bystander effects via soluble SASP factors and extracellular vesicles carrying proteins and regulatory RNAs capable of inducing senescence-like responses in neighbouring cells. In head and neck settings, saliva enables repeated non-invasive sampling; salivary extracellular vesicles are already being explored in oral cancers and may support longitudinal tracking of multiplex SASP-associated modules alongside therapy response and fibrosis development. Overall, these observations underscore the need to incorporate timing and compartmental context into both interpretation and study design. Time-stamping relative to radiotherapy/chemotherapy and pairing liquid biomarkers with spatially resolved tissue analysis offer a plausible route to separate transient injury responses from chronic senescence-linked ECM remodelling.

## 6. Translational Roadmap: Mapping TIS–ECM Niches and Targeting Senescence-Linked Remodelling in HNSCC

### 6.1. Why Translation from Lab to Clinic Is Urgent in Head and Neck Disease

In HNSCC, senescence is rarely an isolated, cell-intrinsic endpoint. Standard-of-care radiotherapy and platinum-based chemotherapy can generate durable senescent states in surviving tumour and stromal compartments, with persistent SASP output that remodels ECM architecture, perturbs perfusion, and reshapes immune access [58]. In the head and neck, these effects carry consequences that extend beyond oncological outcome, because the treated field must maintain complex functions including deglutition, phonation, mandibular opening (trismus-related limitation), salivary gland function, and upper aerodigestive airway protection and patency [154]. HNSCC therefore provides a stringent setting in which to test whether senescence-informed biology can be translated into actionable stratification and intervention, rather than remaining descriptive.

### 6.2. From Single Readouts to Spatially Resolved Senescence–Matrix Phenotyping

A recurring limitation is not the absence of candidate markers, but the difficulty of attributing where senescence sits in tissue, which lineage is affected, and what matrix state accompanies it. Many clinical studies still infer senescence using small immunohistochemical panels (commonly p16 and/or p21 with reduced Ki-67), which can be informative but is often insufficient for lineage-anchored interpretation—particularly in HPV-associated disease where p16 biology is not synonymous with senescence [155,156,157].

A more translationally useful approach is to formalise senescence as a co-localised, spatial “niche” call rather than a bulk property. In HNSCC, this implies:

- Multiplexed senescence panels applied within defined compartments (e.g., fibroblast-rich regions marked by FAP/PDGFRβ/αSMA; endothelial regions marked by CD31), pairing arrest markers with structural/DDR support (e.g., Lamin B1 depletion; persistent DDR foci) and a limited set of ECM-active SASP proteins [1,37,158].
- Quantitative collagen architecture readouts in the same regions of interest, using SHG-derived metrics (alignment, density, organisation) to link senescence localisation to matrix topology that is directly relevant to invasion and immune trafficking [125,159,160].

This is where the signals → matrix → function framework becomes operational: stromal senescent states (signals) broadcast SASP modules that re-engineer collagen organisation (matrix), thereby shaping invasion, immune access, and post-therapy behaviour (function) [68,77,125].

### 6.3. Assigning SASP to the Right Cell, in the Right Place, at the Right Time

SASP measurement is often technically straightforward yet biologically difficult to interpret. Bulk cytokine/protease measurements from tumour lysates, plasma, or saliva can reflect a composite of tumour, stroma, immune infiltrate, infection, and treatment-related injury—limitations that are amplified in the oral cavity, where baseline inflammatory variation and mucosal injury/repair programmes are common.

A defensible translational strategy is therefore to treat SASP as modular and spatially constrained, avoiding inference from single mediators. Therapy-associated SASP programmes can vary by lineage and may evolve with time after treatment [8,151]. A practical next step for HNSCC is to anchor a compact, reproducible ECM-active SASP module (inflammatory mediators plus matrix remodellers/crosslinking-associated proteins) directly to stromal lineages using multiplex imaging, while using fluid-based assays primarily for longitudinal trend monitoring rather than mechanistic attribution [161,162].

### 6.4. Therapeutic Landscape: Senomorphics, Senolytics, and Matrix-Normalising Strategies

If collagen crosslinking and confinement stabilise senescent stromal niches, matrix-normalising strategies that reduce crosslinking and relax confinement could plausibly weaken senescence persistence and diminish chronic ECM-active SASP programmes. Targeting LOX/LOXL activity, for example, may reduce collagen crosslinking, lower stiffness, and increase effective inter-fibrillar spacing, thereby reducing confinement stress and potentially disrupting senescence reinforcement [163,164,165]. Invasion-permissive collagen alignment suggests a complementary intervention point. Limiting the emergence of aligned “tracks” and attenuating adhesion-dependent mechanotransduction could reduce contact-guided tumour migration. Conceptually, this may be approached through strategies that interfere with integrin–FAK signalling or matricellular protein–integrin engagement, particularly where ECM-active SASP programmes promote collagen reorganisation and traction generation [166,167,168]. Importantly, these matrix-directed approaches are mechanistically aligned with senomorphics, which aim to dampen SASP output (including MMPs, LOX/LOXL-associated programmes, and matricellular factors) without necessarily eliminating senescent cells [1,169].

A recurring gap is that ECM mechanics, CAF state, and senescence are often studied in parallel rather than in integrated systems. In HNSCC, analyses that link composite senescence definitions to quantitative collagen architecture (e.g., Second Harmonic Generation-derived alignment and density measures) and spatially resolved stromal phenotyping remain uncommon [32,170]. Addressing this gap will be essential to identify patients harbouring persistent post-therapy senescent–fibrotic niches, and to determine whether combined senescence-modulating and matrix-normalising strategies can be deployed to reduce recurrence risk and/or long-term functional morbidity.

Two broad therapeutic concepts have emerged (Table 2):

Senomorphics (SASP modulators) attenuate harmful outputs of senescent cells without necessarily eliminating them, often via upstream regulators such as mTOR, p38 MAPK, NF-κB, or JAK/STAT signalling [171]. In head and neck tissues, the appeal is that senomorphics may dampen chronic inflammatory and ECM-remodelling programmes while preserving epithelial integrity and wound-repair capacity—key considerations in irradiated mucosa. 

Senolytics (selective clearance) aim to remove senescent cells by exploiting reliance on pro-survival pathways. Across ageing and disease models, senescent cell clearance can improve tissue function and reduce fibrotic phenotypes, but timing and safety are central—particularly in tissues with high microbial exposure and frequent micro-injury such as the oral cavity [172].

**Table 2:** Senotherapeutic classes, mechanisms, and human translation evidence.

Master Class	Sub-Class	Agent	Intended Action	Key Target/Mechanism	Human Translation
**Senomorphics**	Metabolic/inflammatory tone	Metformin	Dampens harmful outputs without cell clearance	Inhibits inflammatory SASP gene expression via IKK and NF-κB interference in senescent cells [173]	MILES trial (NCT02432287) [174]
	mTOR pathway modulation	Rapamycin/rapalogs	SASP attenuation and senescence phenotype re-tuning	mTOR promotes SASP (notably via IL1A translation); rapamycin suppresses SASP outputs [175]	Extensive human exposure in transplantation and oncology supports repurposing feasibility [176]
	JAK–STAT cytokine module suppression	JAK inhibitors (ruxolitinib)	SASP cytokine module suppression	JAK inhibition reduces inflammatory SASP signalling and frailty phenotypes in vivo [66]	Repurposing feasible but chronic use limited by immunosuppression risk [177]
	Stress kinase/NF-κB linked SASP control	p38 MAPK inhibitors	SASP dampening	p38MAPK is a key regulator of SASP independent of canonical DDR signalling [153]	Mixed clinical experience across inflammatory indications [178]
**Senolytics**	First generation small molecule	Dasatinib + quercetin (D + Q)	Clears senescent cells	Identified as senolytic based on senescent cell survival dependencies [179]	Diabetic kidney disease human senolytic biomarker evidence (NCT02848131) [180]
	Skeletal health translation	Dasatinib + quercetin (D + Q)	Clears senescent cells	Same mechanism class as above [181]	Phase 2 RCT in postmenopausal women (NCT04313634) [182]
	HNSCC specific combination	Tislelizumab + D + Q (COIS-01)	Senolysis plus checkpoint blockade	Combines senolysis with immunotherapy in resectable HNSCC [183]	Neoadjuvant study (NCT05724329) [184]
	Flavonoid senolytic	Fisetin	Clears senescent cells in some contexts	Senotherapeutic and senolytic activity in preclinical systems; intermittent dosing rationale [185]	Breast cancer survivorship physical function trial (NCT05595499) [186]
	BCL-2 family	Navitoclax (ABT-263)	Clears senescent cells	BCL-2 and BCL-xL inhibition inducing apoptosis in senescent cells [187]	Systemic use constrained by on-target platelet toxicity; thrombocytopenia documented [188]
	Local/targeted delivery	UBX1325 (BCL-xL inhibitor, ocular)	Local senescent cell clearance	BCL-xL targeting with local delivery to improve therapeutic index [189]	DME clinical trial (NCT04857996) [190]
**Immune-based senolytics**	Senolytic CAR-T	uPAR-targeted senolytic CAR-T	Immune clearance of senescent cells	uPAR is induced on senescent cells; uPAR CAR-T cells ablate senescent cells in vivo [191]	Preclinical: long-term efficacy in aged mice [192]
**Senolytic vaccines**	Senescent immune cell targeting	CD153 vaccination	Immune-mediated clearance of senescent immune cells	Targets CD153-positive senescent T cells; improves metabolic phenotypes in mouse models [193]	Preclinical [193]
	Senescent cell targeting	GPNMB vaccination	Immune-mediated clearance of senescent cells	Vaccination against GPNMB reduces GPNMB-positive senescent cells and improves ageing phenotypes [194]	Preclinical; expanding follow-on work [194]
**Allied anti-fibrotic and matrix normalising**	Anti-TGF-β axis	Anti-TGF-β pathway (class)	Reduces fibrotic reinforcement downstream of SASP	TGF-β is a central mediator of tissue fibrosis and ECM deposition programmes [69]	Multiple agents across indications; oncology translation varies [69]
	Mechanics targeting crosslinking	LOX and LOXL inhibition (class)	Reduces crosslinking and stiffness	LOX family contributes to ECM remodelling and stiffening; therapeutic targeting rationale [73]	LOXL2 inhibition trials in fibrotic disease illustrate translation challenges [73]

In HNSCC, a defensible translational position is that matrix-normalising strategies are mechanistically integral partners to senescence-targeted approaches, because much of the clinically relevant harm is mediated through ECM architecture and mechanics. Anti-fibrotic and matrix-directed approaches (e.g., targeting crosslinking, fibroblast activation programmes, or mechanotransduction pathways) converge on the same endpoints as senotherapies: improved matrix permeability, reduced stiffness-driven exclusionary biology, and attenuation of invasion-permissive collagen alignment [195]. A pragmatic logic is sequencing:

- Early post-therapy windows may favour senomorphic approaches combined with matrix normalisation to limit consolidation of a stiff, aligned, senescence-reinforcing niche [196,197].
- Later windows, where persistent fibrotic stroma is established, represent the context in which senolytic concepts are most testable—provided safety monitoring explicitly addresses mucosal integrity and infection risk [123,196,198].

From a translational perspective, the clinical picture is encouraging, although it remains uneven across senomorphics, senolytics, and matrix-normalising strategies. Senomorphics currently offer the most immediate repurposing potential because several candidate agents already have extensive human exposure in other indications. Metformin has been shown to interfere with NF-kB-dependent SASP programmes in senescent cells, and human ageing-focused studies support biologic activity on ageing-associated metabolic and non-metabolic pathways, consistent with a senomorphic rationale rather than direct senescent-cell clearance [173,199]. mTOR-directed approaches are also attractive in this setting, as mTOR promotes pro-inflammatory SASP output, including IL1A-dependent reinforcement, whereas rapamycin suppresses these outputs experimentally [175]. Likewise, JAK inhibition can dampen inflammatory SASP signalling [66], while p38 MAPK remains mechanistically relevant as a regulator of SASP outside canonical DDR signalling [153]. Collectively, these data support senomorphics as plausible adjunctive approaches where modulation of chronic inflammatory and matrix-active programmes is desirable, particularly in tissues where preservation of repair capacity may be advantageous.

Clinical translation of senolytics is also beginning to mature. Intermittent dasatinib plus quercetin (D + Q) has advanced into randomised human testing, and a phase 2 trial in postmenopausal women showed that senolytic therapy can be delivered on an intermittent schedule, with exploratory evidence suggesting that responses may be greatest in individuals with a higher baseline senescent-cell burden [182]. This observation is especially relevant to translational oncology, where biomarker-enriched deployment may prove more informative than unselected administration. In HNSCC, this principle aligns well with the possibility that composite, spatially anchored senescent-fibrotic niches may eventually help identify patients most likely to benefit from senolytic intervention.

A disease-relevant signal has also begun to emerge in HNSCC itself. The COIS-01 programme combines neoadjuvant tislelizumab with dasatinib and quercetin in resectable HNSCC, reflecting growing interest in pairing senolysis with immunotherapy. Published early-phase results reported a major pathological response rate of 33.3%, with a low incidence of grade 3–4 adverse events, supporting the feasibility of this combination and suggesting that senolytic-immunotherapy strategies may have translational potential in selected cancer settings [183,184]. Continued oncology-phase development is also evident in related contexts, including an active phase II fisetin trial in breast cancer survivors and an active early-phase glioma study combining dasatinib, quercetin, fisetin, and temozolomide [186,200].

Additional senolytic agents further support the view that this is an expanding therapeutic space. Fisetin remains attractive because of its intermittent senotherapeutic rationale and comparatively accessible translational profile, and it is being evaluated in breast cancer survivorship with the aim of improving physical function after chemotherapy [185,186]. Navitoclax is also relevant in this context. In relapsed or refractory lymphoid malignancies, navitoclax demonstrated antitumour activity in a subset of patients, indicating that BCL-2/BCL-xL-directed strategies are clinically tractable [188]. At the same time, its development has highlighted a central challenge of systemic senolysis, namely on-target thrombocytopenia related to BCL-xL inhibition. In this respect, localised or compartment-restricted approaches may help improve therapeutic index. UBX1325 is informative here: in diabetic macular oedema, this locally delivered BCL-xL inhibitor showed good tolerability and efficacy signals sufficient to support further evaluation, suggesting that spatially restricted senolytic delivery may widen the therapeutic window of this class [189,190].

Immune-mediated senolytic strategies remain preclinical, but the available data are notable. uPAR-targeted senolytic CAR-T cells were shown to ablate senescent cells in vivo and reverse senescence-associated pathologies in mouse models, including fibrosis-associated settings [191,192]. Vaccine-based approaches point in a similar direction. CD153 vaccination reduced adipose senescent T-cell accumulation and improved metabolic phenotypes in obese mice [193], whereas GPNMB-directed senolytic vaccination reduced GPNMB-positive senescent cells, improved age-related phenotypes, reduced atherosclerotic burden, and extended lifespan in progeroid male mice [194]. Although these strategies remain preclinical, they illustrate that immune-mediated senolysis can be biologically meaningful, durable, and potentially adaptable to chronic post-therapy senescent niches.

Matrix-normalising approaches may be particularly relevant in the present framework because they target not only the senescent cell, but also the senescence-supportive niche. Here, the translational evidence is mixed but informative. TGF-β pathway inhibition has entered oncology trials; galunisertib plus gemcitabine improved overall survival relative to gemcitabine alone in unresectable pancreatic cancer, with minimal added toxicity [201]. LOX/LOXL targeting has also reached the clinic. Simtuzumab, a LOXL2-directed antibody, was well tolerated in metastatic pancreatic cancer but did not improve outcomes when added to gemcitabine, and it likewise failed to show efficacy in idiopathic pulmonary fibrosis [202,203]. These findings suggest that matrix-normalising strategies are clinically tractable and biologically well grounded, but that their benefit is likely to depend strongly on timing, disease context, biomarker selection, and a combinatorial partner. In parallel, broader stromal-normalising experience in oncology supports the principle that matrix-directed intervention can be therapeutically useful; for example, total neoadjuvant therapy including losartan in locally advanced pancreatic cancer was associated with substantial downstaging and an R0 resection rate of 61%, consistent with the idea that matrix-remodelling interventions can improve treatment delivery and tumour control in selected settings [204].

Overall, the field is now sufficiently advanced to support cautious optimism. Clinical studies indicate feasibility, early efficacy signals are beginning to emerge, and the first HNSCC-specific combination data are now available. The clearest near-term opportunity is likely to come not from asking whether senotherapeutics work in general, but from identifying which post-therapy niche is present, when it is most vulnerable, and which intervention is best matched to that niche: SASP attenuation, selective senescent-cell clearance, matrix relaxation, or rational combinations of these. In HNSCC, where persistent fibrosis, immune exclusion, and survivorship morbidity are central concerns, this supports biomarker-guided combination development rather than one-size-fits-all deployment.

### 6.5. A Workable Biomarker Framework for Trials: The Composite TIS–ECM Score

**Figure 5:**
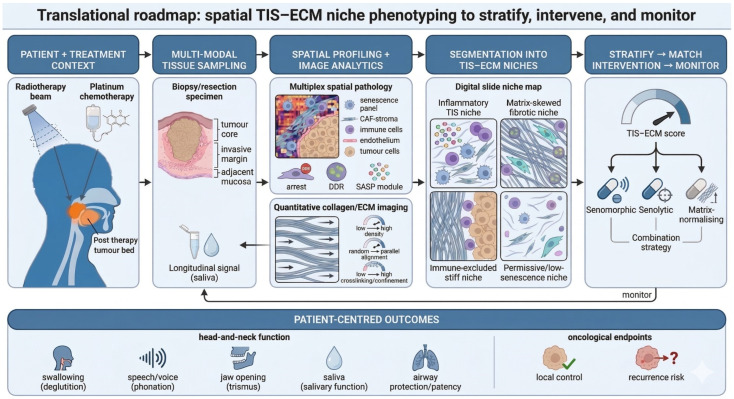
Translational roadmap for spatial TIS–ECM niche phenotyping to stratify, intervene, and monitor in HNSCC. Schematic workflow linking routine treatment exposures (radiotherapy and platinum-based chemotherapy) to therapy-induced senescence (TIS) and downstream extracellular matrix (ECM) remodelling in head and neck squamous cell carcinoma (HNSCC). Patient and treatment context motivates multi-modal, time-stamped tissue and liquid sampling (e.g., biopsy/resection across tumour core, invasive margin, and adjacent/remote mucosa, alongside longitudinal saliva). Spatial profiling combines multiplex pathology to localise senescence within defined compartments (e.g., CAF-rich stroma, endothelium) using a multi-axis readout (arrest markers with structural/DNA-damage response support and a compact, ECM-active SASP module), together with quantitative collagen/ECM imaging (e.g., SHG-derived density, alignment, and organisation metrics). These integrated data are segmented into interpretable TIS–ECM “niches” (e.g., inflammatory vs. matrix-skewed/fibrotic; immune-excluded vs. permissive), enabling computation of a tissue-anchored TIS–ECM score for patient stratification, rational matching to senomorphic, senolytic and/or matrix-normalising interventions, and longitudinal monitoring. Patient-centred outcomes emphasise both oncological endpoints and head-and-neck function (swallowing, phonation, trismus/jaw opening, salivary function, airway protection/patency).

To connect mechanism to patient stratification, composite readouts are more realistic than any single marker. A TIS–ECM score provides an implementable framework because it is tissue-anchored, multi-modal, and suited to longitudinal sampling. In practical terms, the score can integrate:

- Senescence burden: Co-localised arrest plus structural/DDR support within defined stromal/endothelial compartments.
- SASP modules: A compact panel including inflammatory mediators plus ECM-active factors, interpreted with explicit timing relative to therapy.
- Matrix topology/mechanical proxies: SHG-derived collagen density/alignment/organisation, ideally focused on invasive fronts and post-treatment margins [159] (Figure 5).

This creates a shared pharmacodynamic language to test whether an intervention reduces senescence burden, shifts SASP composition away from chronic matrix remodelling, and/or measurably changes collagen architecture towards less restrictive, less invasion-permissive states. In concrete terms, the TIS–ECM score can be operationalised using the following variables: (i) a senescence burden score (semi-quantitative, 0–3) assigned within CAF-rich regions, based on co-localised p16/p21 positivity with Ki-67 absence plus at least one structural/DDR hallmark (Lamin B1 depletion, persistent γH2AX/53BP1 foci interpreted ≥6 weeks post-therapy, or GLF16 lipofuscin signal); (ii) an ECM-active SASP module score incorporating MMP and SERPINE1/PAI-1 expression co-localised within the same stromal compartment; and (iii) a collagen architecture index derived from SHG-based alignment, density, and organisation metrics at invasive fronts and post-treatment margins. Two implementation tiers can be distinguished. A routine pathology tier, implementable with existing infrastructure, would comprise a limited IHC panel (p16, p21, Ki-67, Lamin B1, αSMA or FAP for lineage assignment) combined with semi-quantitative collagen scoring using picrosirius red under polarised light. A research/trial tier would add multiplex spatial pathology with GLF16, quantitative SHG microscopy, and spatial transcriptomics-derived senescence scores (e.g., DeepScence, hUSI). This tiered structure ensures the core framework is accessible for hypothesis testing in routine clinical material, while the full spatial-omics version provides the resolution needed for mechanistic inference. For clinical trial enrichment, the score could stratify patients into “senescence-high/matrix-stiff” versus “senescence-low/matrix-permissive” groups, with the former hypothesised to derive the greatest benefit from combined senomorphic and matrix-normalising intervention. Threshold definitions will need to be empirically derived in pilot cohorts, but the COIS-01 observation that senolytic benefit may be greatest in individuals with higher baseline senescent-cell burden supports the biomarker-enrichment logic [182,183,184]. The composite score should be evaluated both as a baseline stratification tool and as a pharmacodynamic endpoint measuring on-treatment change, providing a shared language for sequential senomorphic, senolytic, and matrix-normalising trials.

## 7. Conclusions

This review has stated that senescence in HNSCC is best understood as a tissue-organised process in which senescent stromal states, their secretory outputs, and the extracellular matrix are bidirectionally coupled. The signal → matrix → function framework proposed here captures this reciprocity: SASP modules drive ECM remodelling (collagen architecture, i.e., deposition, crosslinking and alignment), while the remodelled matrix in turn stabilises senescent niches through mechanotransduction and confinement. In HNSCC, this coupling has direct consequences for both late treatment toxicity—including fibrosis, xerostomia, and dysphagia—and for recurrence-relevant biology, because stiffened, aligned collagen tracks may simultaneously sustain senescent stroma and facilitate invasion.

Key priorities for the next phase of the literature include: (i) longitudinal, time-stamped studies that distinguish early injury responses from persistent post-therapy niches; (ii) standardised, HNSCC-appropriate composite senescence definitions that account for HPV-associated p16 biology and high baseline inflammatory tone; and (iii) routine integration of quantitative ECM phenotyping—collagen density, alignment, and organisation—alongside senescence localisation, so that the field can progress from association to mechanism.

The composite TIS–ECM score proposed in Section 6.5 offers a practical pharmacodynamic framework for clinical trials, combining compartment-anchored senescence burden, modular SASP readouts, and quantitative matrix topology into a shared language for evaluating senomorphic, senolytic, and matrix-normalising interventions. Translating this framework from concept to clinical utility will require coordinated investment in spatial pathology, longitudinal tissue access, and cross-disciplinary collaboration between senescence biologists, matrix scientists, and head and neck clinicians.

## Figures and Tables

**Table 1:** SASP categories, representative factors, and therapeutic targeting.

SASP Category	Representative Factors	ECM/TME Consequence	Existing Drugs/ Therapies	Status/Notes	Key References
**Cytokines/Chemokines**	IL-6, IL-8, TNF-α, CXCL1, CCL2	Chronic inflammation, CAF activation, angiogenesis, immune evasion	JAK/STAT inhibitors (ruxolitinib, baricitinib), anti-IL-6 (tocilizumab)	Approved for inflammatory disease; limited oncology use	[6,7,8,60,61,62,64,65,66]
**Growth Factors**	TGF-β, VEGF, HGF	Fibrosis, angiogenesis, EMT, tumour cell plasticity	TGF-β inhibitors (galunisertib, fresolimumab), VEGF inhibitors (bevacizumab, aflibercept)	Oncology trials; some discontinued due to toxicity	[6,7,60,67,68,69]
**Proteases**	MMP-1, MMP-3, MMP-9, uPA, cathepsins	ECM degradation, invasion tracks, metastasis	Broad MMP inhibitors (batimastat, marimastat), uPA inhibitors	Most early MMP trials failed due to off-target toxicity	[6,8,11,47,60]
**Crosslinkers and Matricellular Proteins**	LOX/LOXL, POSTN, FN1, THBS1	Collagen crosslinking, stiffening, alignment, invasion scaffolds	LOXL2 inhibitors (simtuzumab, PXS-5505), anti-POSTN antibodies (pre-clinical)	Antifibrotics in early phase; PXS-5505 in myelofibrosis	[11,70,71,72,73]
**Lipids**	Prostaglandins, leukotrienes, oxidised lipids	Pro-inflammatory milieu, endothelial activation, barrier dysfunction	COX inhibitors (NSAIDs, celecoxib), leukotriene antagonists	Approved anti-inflammatory; repurposing explored in cancer	[6,8,60]
**Extracellular Vesicles (EVs)**	EV-bound cytokines, miRNAs, matrix enzymes	Long-range SASP signalling, ECM remodelling, niche conditioning	No direct EV therapies; targeting EV biogenesis (e.g., GW4869)	Pre-clinical, conceptual stage	[43,44]

## Data Availability

This study did not involve the generation or analysis of any datasets.

## Abbreviations

The following abbreviations are used in this manuscript:

SASP	Senescence -associated secretory phenotype
ECM	Extracellular matrix
HNSCC	Head and neck squamous cell carcinoma
CAF	Cancer-associated fibroblast
TIS	Therapy-induced senescence
HPV	Human Papilloma Virus
